# Acyclovir-resistant chronic mucocutaneous herpes with good response to the association with imiquimod in an AIDS patient: case report^⋆^

**DOI:** 10.1016/j.abd.2020.10.019

**Published:** 2022-01-14

**Authors:** Suellen Ramos de Oliveira, Ariane Sponchiado Assoni, Thiago Jeunon de Sousa Vargas, Egon Daxbacher

**Affiliations:** Hospital Federal de Bonsucesso, Rio de Janeiro, RJ, Brazil

Dear Editor,

Genital ulcers are often caused by herpes simplex virus (HSV), with type 2 being most frequently implicated.^1^ When immunodeficiency is present, the herpetic infection can progress with atypical presentations and greater refractoriness to antiviral treatment.^2^

In patients who are co-infected with human immunodeficiency virus (HIV), deeper ulcerations, hypertrophic lesions, and, in some cases, lesions with pseudotumor characteristics may be seen.^1^

This case report describes a patient with acquired immunodeficiency with anogenital lesions, initially resistant to treatment with systemic acyclovir, who showed significant improvement after its association with the topical use of 5% imiquimod cream.

A case of a 38-year-old female patient with acquired human immunodeficiency syndrome (AIDS) diagnosed approximately five years ago, but who showed loss of adherence to antiretroviral treatment (ART) for a few months during 2017, restarting treatment in December of that same year is presented. Among the laboratory alterations, she had a CD4 T-cell count of 4 cells/μL. She reported the presence of ulcerated anogenital lesions for approximately one year. She had been medicated at another service with oral acyclovir for four months, without any improvement.

At the time of the examination, she had extensive, intensely painful, ulcerated lesions with crusts in the vulva and perianal region (Fig. 1a). Intravenous acyclovir was the drug of choice, at a dose of 10 mg/kg/dose 3 times a day, associated with 5% imiquimod cream (3 times/week), with excellent tolerability. She showed a good therapeutic response three weeks after beginning the proposed treatment (Fig. 1b), but it could not be concluded as the patient died due to complications from the underlying disease. The histopathological examination was carried out at the beginning of the clinical picture in order to document the case, showing the typical findings of HSV infection (Fig. 2).

**Figure 1:**
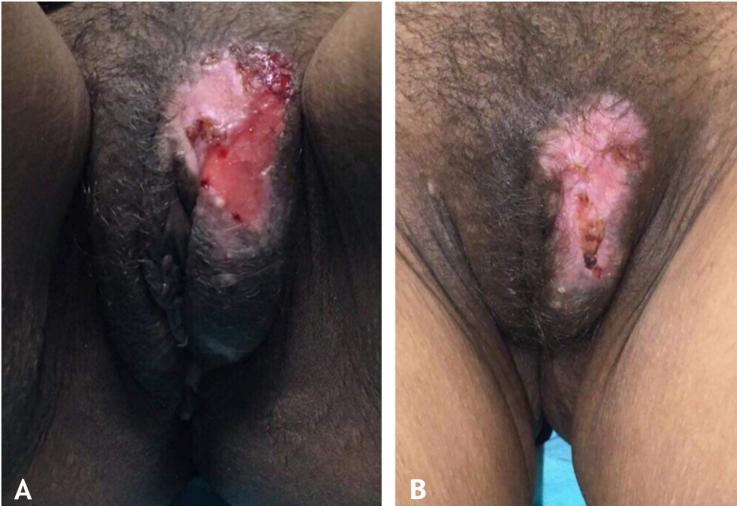
(A), Extensive ulcer with crusts in the vulva. (B), Cicatricial aspect of the lesion after association with 5% imiquimod cream.

**Figure 2:**
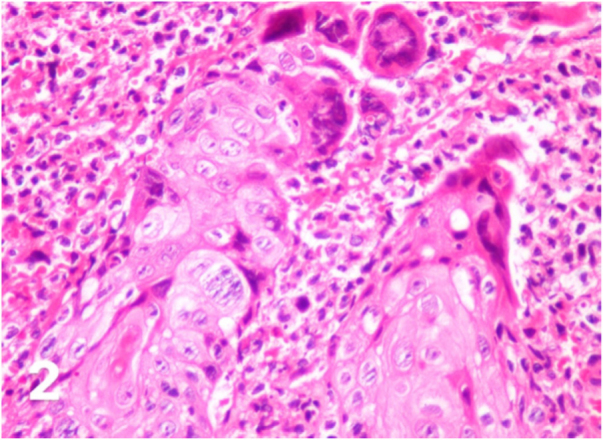
Cytopathic effects of herpes simplex such as ballooning degeneration of keratinocytes, marginalized chromatin, multinucleated giant cells (Hematoxylin & eosin, ×400).

It is believed that unusual clinical presentations due to HSV infections in AIDS patients could be explained as a reflection of the slow evolution of the disease and not necessarily due to the specific characteristics of the involved strains.

Imiquimod is an immunomodulator with a formal indication for the topical treatment of conditions such as external anogenital warts, superficial basal cell carcinoma, and actinic keratoses.^3^ Its action is due to its antiviral and antitumor effects.^4^ Although not considered a formal indication of this medication, the antiproliferative action of imiquimod has shown significant importance in the adjuvant treatment of genital ulcers caused by HSV, particularly those refractory to conventional treatment, such as in our patient, who had already been treated with long-term oral acyclovir.

There is a report in the literature, as early as 2002, indicating the successful use of topical imiquimod for the treatment of genital ulcers in an HIV-positive patient.^5^ More recently, two other cases have been reported, both presenting clinical resistance to conventional antiviral treatment, which had excellent results with the association of topical imiquimod.^6^ There are descriptions in the literature of significant improvement of the lesions after the sixth day of use of the association of imiquimod with the traditional treatment.^7^

Foscarnet sodium, in cases of ulcers that are resistant to acyclovir, has been the drug of choice for the associated treatment. However, it is known that there are drug-resistant strains that replicate even during its use.^8^ Despite not having undergone a viral resistance test to acyclovir, the patient in the present case report had been submitted to monotherapy with this medication for months without any improvement. Studies of resistance patterns of HSV to different antiviral agents used in daily practice and the characterization of mutant strains of HSV have shown that resistance is due to loss or modification of the viral enzyme thymidine kinase or changes in viral DNA polymerase.^9^ Based on the promising results of the association of topical imiquimod with the established antiviral therapy, including cases that were not submitted to drug susceptibility testing, the combination therapy was chosen. It is believed that the improvement in local immunity provided by imiquimod would explain the clinical improvement of the lesions, as the increase in interferon alpha plays an important role in controlling viral infections.^7^ Considering the relatively rapid response to the proposed treatment, the hypothesis that this would not be a case of drug resistance, but refractoriness to the treatment due to deficient local immunity in a patient with AIDS is supported.

There have been reported cases of occlusion therapy with this topical drug, a technique that is usually not well accepted by patients due to local side effects. However, a study was published in which the patient showed an excellent result and no side effects, with complete regression of the lesion after 10 weeks of imiquimod use.^10^

Finally, it is important to emphasize that lesions with unusual presentations can make the diagnosis of HSV difficult. The main therapeutic regimen remains the use of acyclovir, but in patients with immunodeficiency, the association of the antiviral and the topical use of imiquimod has shown good results. Although further studies are still required, the case reported herein may help to support the use of imiquimod as a promising drug in the treatment of genital infections caused by HSV refractory to the traditional therapy.

## Financial support

None declared.

## Authors’ contributions

Suellen Ramos de Oliveira: Concepts; design; definition of intellectual content; literature search; clinical studies; data acquisition; data analysis; manuscript preparation; manuscript editing; manuscript review; guarantor.

Ariane Sponchiado Assoni: Concepts; design; definition of intellectual content; literature search; clinical studies; data acquisition; manuscript preparation; manuscript editing; guarantor.

Thiago Jeunon de Sousa Vargas: Concepts; design; definition of intellectual content; clinical studies; data analysis; manuscript review; guarantor.

Egon Daxbacher: Concepts; design; definition of intellectual content; clinical studies; data analysis; manuscript review; guarantor.

## Conflicts of interest

None declared.
